# Impact of a Rapid Assessment Zone on Patient Throughput in an Urban Pediatric Emergency Department

**DOI:** 10.5811/westjem.62068

**Published:** 2026-08-06

**Authors:** Amanda Schoonover, Alyssa M Gill, Emily E Hill, Zina Alhaddad, Jessica Parker, Jackson Lanphear, Bhawana Arora

**Affiliations:** * Michigan State University College of Human Medicine, Grand Rapids, Michigan; † Corewell Health/Helen DeVos Children’s Hospital, Pediatric Emergency Department, Grand Rapids, Michigan; ‡ Corewell Health Research Institute, Department of Statistics, Grand Rapids, Michigan

## Abstract

**Introduction:**

The rapid assessment zone is a newer model for optimizing emergency department (ED) throughput. Similar models implemented in adult ED settings have shown improvements in throughput parameters. However, few studies have taken place in the pediatric ED setting. Our study objective was to assess the impact of implementing a rapid assessment zone on throughput in an urban pediatric ED. We hypothesized that length of stay (LOS), time to bed placement, and time to see a clinician would decrease after the intervention and remain sustainable over long-term follow-up.

**Methods:**

This retrospective study assessed the effect of rapid implementation from May 2012–April 2018 on LOS for ED visits for three two-year time periods: pre- and post-rapid assessment zone and post-follow-up. Our primary outcome measures were LOS, time from registration to bed, and time from registration to first clinician evaluation. Secondary outcome measures were how LOS differed between high- and low-acuity visits. We analyzed data using Kruskal-Wallis for comparison of medians and Wilcoxon rank-sum tests with Bonferroni correction for pairwise comparisons.

**Results:**

There were 304,318 unique ED visits from May 1, 2012–April 30, 2018, with an increase in volume over time (92,551 visits pre-rapid assessment zone and 106,530 visits post-follow-up). Overall median LOS decreased from pre- rapid assessment zone, post- rapid assessment zone, and post-follow-up, respectively (110 minutes versus 101 minutes vs 94 minutes; *P* < .001). When stratified by acuity level, low-acuity visit LOS decreased from pre- rapid assessment zone to post- rapid assessment zone to post-follow-up (85 min vs 77 min vs 70 min; *P* < .001), whereas LOS for higher-acuity levels was, on average, unchanged (144 min vs 149 min vs 143 min for pre-RAZ, post- rapid assessment zone, and post-follow-up, respectively; *P* < .001). The overall registration to clinician time decreased from 15 minutes to 13 minutes (*P* < .001) between pre-rapid assessment zone and postfollow-up, which is statistically but not clinically significant.

**Conclusion:**

Implementation of a rapid assessment zone significantly decreased overall length of stay in our pediatric ED despite increased patient volume, especially for lower acuity patients. The rapid assessment zone model can be effective in preventing crowding in a pediatric ED with a large proportion of low-acuity visits.

## INTRODUCTION

Optimization of emergency department (ED) front-end operations has become a crucial area of focus for quality improvement because crowding has been associated with negative effects on patient health and well-being.^1^–^5^ Examples of front-end–focused methods that have been shown to be effective include physician-led triage and split-flow models, including fast-tracking lower acuity patients.^6^ One specific model for optimizing throughput is the rapid assessment zone, which streamlines lower-acuity patients to designated areas (“zones”) for joint assessment and disposition by ED nurses and clinicians. While lower acuity visits typically require minimal care, they disproportionately contribute to ED crowding, which impacts the care of all patients.^7^

Rapid assessment zones or related models have been implemented and studied in adult EDs, with most showing modest improvement in time-related patient outcomes.^8^–^12^ However, there are few reported studies of the impact of rapid assessment zones, or similar throughput modification, on similar outcomes in pediatric EDs (PED).^13^–^16^ Pediatric EDs experience higher rates of low-acuity visits due to a low threshold for ED visits for childhood illness, despite having a primary care physician.^17^ This results in longer wait times, delayed assessment and disposition processes, and high rates of left without being seen. Therefore, efficiency in managing low-acuity visits is vital for reducing crowding and improving patient outcomes for PED visits of all acuity levels.

At the time, our study was the first to describe the implementation of a rapid assessment zone in an urban PED and its effect on time-related outcomes. Prior to the implementation of the. rapid assessment zone, patients were equally divided on arrival into one of two ED zones regardless of acuity or concern, except for patients requiring resuscitation. The new model, however, allows for 60% of visits to be streamlined to the rapid assessment zone for efficient evaluation and improved movement through the ED. When operating at full capacity, the rapid assessment zone has 15 beds, with five to seven focused on initial assessment of the patient. Staffing changes throughout the day to account for patient arrival volumes. The area is staffed by one attending physician, one to two nurse practitioners or physician assistants, one to two resident physicians, and two to four nurses, with maximum staffing during peak arrival times.

In this model, patients who present to the ED are initially triaged by nurses who determine patient allocation based on acuity and anticipated length of time needed for patient care. Low-acuity patients with anticipated LOS of < 60 minutes are sent to the rapid assessment zone, with further distribution based on expected diagnostic and therapeutic needs. Moderate- and high-acuity patients, or those with anticipated high-resource use with LOS > 60 minutes are sent to the traditional ED track. A list of inclusion and exclusion criteria is available for triage nurses to aid in determining the disposition of the patient (Table 1). Rapid assessment zone allocation is followed by joint assessment and disposition by a physician and nurse, expediting assessment and diagnosis in this new process. The rapid assessment zone inclusion and exclusion criteria have been modified since their implementation. Originally, inclusion criteria were limited to quick disposition and short LOS. However, this practice overwhelmed the other patient care areas due to few patients meeting criteria, and due to lack of objective parameters for rapid assessment zone allocation. Over time, additional complaints such as specific types of abdominal pain, chest pain in noncardiac patients, or nonpetechial rashes can be considered for the rapid assessment zone (Table 1). Additional details of the model, including its development and implementation, are published elsewhere.^18^

**Population Health Research Capsule**

What do we already know about this issue?

*Front-end focused methods of optimizing emergency department (ED) throughput are effective in adult EDs. Pediatric EDs experience crowding due to their high volume of low-acuity visits*.

What was the research question?

*Does throughput in an urban pediatric ED improve with the implementation of a rapid assessment zone?*

What was the major finding of the study?

*Median length of stay decreased 16 minutes (15%; 110 min to 94 min; P < .001) and 18% for low-acuity visits*.

How does this improve population health?

*Optimizing pediatric ED throughput with front-end facing methods like rapid assessment zones promotes improved quality of care and the ability to care for more patients*.

The aim of this study is to add to the paucity of literature on the impact of rapid assessment zone implementation on PED throughput. Improved throughput with rapid assessment zone implementation is expected due to the accelerated triage care, elimination of upfront registration tasks, immediate placement in ED beds, and joint physician/nurse assessment, all of which streamline movement through the ED. We hypothesize that the LOS, time to bed placement, and time to see a clinician will decrease post- rapid assessment zone implementation and will sustain post-follow-up. Furthermore, we expect that this change will improve LOS for lower acuity visits, without worsening LOS for moderate- and high-acuity visits.

## METHODS

This retrospective, before-and-after study assessed the effect of rapid assessment zone implementation on an urban PED throughput. Consecutive patient visits were collected from the electronic health record (EHR) for a 6-year period and then separated into 2-year time periods to compare throughput outcomes from before implementation of a rapid assessment zone to the two years after, and the following two years for sustained effect. Visits were collected from May 1, 2012–April 30, 2018. Data were then divided into three groups based on 2-year time frames: pre- rapid assessment zone (May 1, 2012–April 30, 2014); immediately post-rapid assessment zone (May 1, 2014–April 30, 2016); and post-follow-up (May 1, 2016–April 30, 2018). Time-related outcome data exported from the EHR included precalculated time from arrival registration to seeing a clinician (registration to clinician), time from registration to placement in a bed (registration to bed), and time from registration to discharge ie, ED LOS. We excluded the visits where the data were missing, partial, or not logical, such as no acuity documented; multiple dates/times; LOS < 0 minutes or > 5 days; time from registration to bed or clinician greater than LOS; left without being seen and saw a clinician; and time from registration to clinician or bed being < 0 minutes.

The main outcomes assessed were ED LOS, time from registration to placement in a bed, and time from registration to seeing a clinician. To examine how rapid assessment zone implementation affected these ED throughput parameters, we assessed for trends in LOS over the study period as well as comparing averages in LOS, registration to bed, and registration to clinician, between the three time periods (pre- and immediately post-rapid assessment zone, and post-follow-up). Additionally, we examined how overall LOS differed between high-to-moderate acuity (levels 1–3) and low-acuity (levels 4 and 5) visits over the study period.

All time-related outcomes were in terms of minutes and treated as continuous variables. Data were not normally distributed and, therefore, were thus analyzed with Kruskal-Wallis for nonparametric comparisons of statistical difference and reported as median (interquartile range [IQR]: 25^th^, 75^th^ percentile). If the medians were significantly different across the three time periods, we used Wilcoxon rank-sum tests for pairwise comparisons with Bonferroni correction for reducing type 1 error. All analyses were conducted using SAS software v9.3 (SAS Institute Inc, Cary, NC). *P* values < 0.05 were considered significant.

Our methods adhered to most of the Worster and Bledsoe (2005) methodologic standards in medical record review. Specifically, data abstractors were trained and used an abstractor form, sampling methods were described (which included using medical records), and case selection criteria were developed with specific variable definitions.^19^ We did not, however, blind the abstractor to the details of the study or discuss interobserver reliability. Missing data were simply excluded and not managed statistically. This study was considered exempt by the institutional review board.

## RESULTS

There were 304,318 unique ED visits from May 1, 2012–April 30, 2018, after excluding 5,147 visits. Visits were divided into pre- rapid assessment zone (May 1, 2012–April 30, 2014; n = 92,551), post-rapid assessment zone (May 1, 2014–April 30, 2016; n = 105,237), and post-follow-up (May 1, 2016–April 30, 2018; n = 106,530) (Figure 1). Volume increased over time (92,551 visits pre-rapid assessment zone to 106,530 visits post-follow-up). High-to-moderate acuity level visits were more prevalent in the pre- rapid assessment zone rapid assessment zone time as compared to post-rapid assessment zone and post-follow-up (51.8% versus 42.4% versus 42.8%).

Overall median ED LOS decreased by 14.5%; from 110 minutes (IQR 72–165) pre-rapid assessment. zone to 101 minutes (64–156] post-rapid assessment zone, and to 94 minutes (59–148) post-follow-up (*P* < .001). We did not find clinically significant differences in length of time from registration to bed after the implementation of rapid assessment zone. Time from registration to clinician contact decreased from 15 minutes pre-rapid. assessment zone to 13 minutes post-follow-up (*P* < .001); however, this difference of 2 minutes is likely not clinically significant (Table 2).

When stratified by acuity level, we found that median ED LOS (minutes) for low-acuity visits significantly decreased over the study periods from pre-rapid assessment zone, to post-rapid assessment zone, and then to post-follow-up (85 versus 77 vs 70; *P* < .001). This corresponds to a 17.5% reduction in average LOS for lower acuity visits from pre-rapid assessment zone to post-follow-up. For high-to-moderate-acuity level visits, LOS was, on average, unchanged (144 min vs 149 min vs 143 min for pre-rapid assessment zone, post-rapid assessment zone, and post-follow-up, respectively; *P* < .001) (Figure 2).

When LOS was stratified by month for evaluation of seasonal variation, peaks in visit time varied by month and year, and these changes were similar between low- and higher acuity groups (ie, both acuity groups reflected a similar magnitude of increase in LOS when an increase was observed) (Figure 3). From January to March in 2013, 2015, and 2018, more dramatic increases in LOS were seen. Despite seasonal upticks in LOS, lower acuity visits still trended downward while higher acuity visits remained minimally changed.

## DISCUSSION

We found that the implementation of a rapid assessment zone improved throughput in an urban PED. We noticed a significantly decreased median LOS after rapid assessment zone implementation by 16 minutes (14.5%; *P* < .001). While performed in adult EDs, studies conducted at other institutions have shown data consistent with these findings.^7^–^12^ A 2005 study found that average monthly wait times decreased by 24 minutes (95% CI, 10–38), and overall ED LOS decreased by 31 minutes (95% CI, 6–57) after implementing their rapid triage model.^10^ Similarly, White et al (2012) implemented a “supplemented triage and rapid treatment” model resulting in decreased overall LOS by 29 minutes (from 361 minutes to 332 minutes; *P* < .001).^8^ A more recent study by Anderson et al (2020) also found that implementation of a rapid assessment zone rapid assessment zone reduced median LOS by 31 minutes (15.8%), and also time from arrival to clinician contact decreased by 15 minutes (53.6%).^12^ Differences in results may be due to a lack of a standard outcome, differences between pediatric and adult EDs in staffing, patient volume and relative acuity, and registration needs.^20^

Few have studied the effect of a rapid assessment zone or other throughput optimization models on outcomes in PEDs. However, a study quite similar to ours was very recently published and reflected the same findings, including a decreased LOS of 15.7 minutes (*P* < .001) compared to our 16 minutes.^21^ The main differences between the studies are that we placed a focus on stratifying our data by acuity, whereas they placed a focus on the rate of left without being seen, which was excluded from our study. In general, a recent technical report by the American Academy of Pediatrics summarizes the available evidence and suggests guidelines to improve PED crowding.^17^ Throughput-related solutions included staffing and room allocation models such as fast tracks or streaming, rapid assessment zone, and clinician in triage. However, given the lack of available data, most of the cited studies were conducted in adult EDs; therefore, the results may vary when compared with PEDs as seen in our study and Ramirez et al.^21^ A systematic review by Morley et al also provides a comprehensive summary on the different interventions aimed at optimizing ED throughput and their associated outcomes.^6^ This includes not only front-end methods such as a rapid assessment zone but also other effective strategies, such as bed management and point-of-care testing.

We also found that the overall reduction in LOS differed by patient acuity, with those at lower levels of acuity benefiting the most with a more sustained and larger decrease in LOS over time. This trend was observed despite seasonal LOS upticks during upper respiratory illness months. This finding was expected given the nature of lower acuity concerns necessitating fewer tests and less complex or time-consuming interventions. The overall reduction in LOS was likely due to increased bed availability/turnover and, therefore, shorter wait times for all patients. One study similarly found that their intervention decreased LOS among lower acuity visits with no significant change in LOS for high- to moderate-acuity visits. The same group then published another paper, detailing how they further modified their model to successfully improve LOS specifically for high-acuity patients.^22^ Importantly, the modifications were cost-neutral (ie, they did not require additional clinicians, space, or resources). These results, albeit conducted in an adult ED, are promising and may represent a future of iterative improvements in throughput optimization models to suit the unique needs of PEDs and benefit all acuity patients.

We expected this benefit in LOS for lower acuity visits, but questioned whether LOS would change for higher acuity visits. We observed no clinically significant change or worsening in LOS over time for high-acuity visits, although the volume of high-acuity visits decreased approximately 10% over the study period, which may have affected the results. This finding is, however, consistent with available literature, which has demonstrated that throughput models similar to the rapid assessment zone have not had a negative impact on high-acuity visit LOS or delay in first point of contact with a physician.^23^ Further modification to the rapid assessment zone protocol, similar to the work of Chartier et al could be conducted in an attempt to decrease LOS among high- to moderate-acuity visits, as well.

The consistency in data among multiple institutions of varying settings, patient populations, and ages—including data from our PED—aids in bridging the gap in generalizability that was previously present in the data pool. The improvement in LOS as shown in our study can potentially improve patient satisfaction, bed availability in times of patient surges, bed availability to critically ill and injured patients, and staff utilization. Joint assessment with the physician and nursing staff is not only time effective but also aids in collaboration with the multiple members of the care team. This collaboration can improve patient care, communication, and satisfaction. Other secondary outcomes, such as patient satisfaction, have been shown to improve with rapid assessment zone implementation in multiple studies conducted in adult EDs.^1^,^24^,^25^ While we did not assess patient satisfaction with our data collection, we recognize the importance of considering the up- and down-stream effects of rapid assessment zone implementation on all patients. Future research is warranted to identify and examine the negative and positive externalities associated with implementing such models and how to mitigate or accentuate those effects.

The effect of implementing a rapid assessment zone on ED throughput may differ in larger cities or more rural areas with different patient populations and, therefore, acuity. In 2018, over 55% of visits to our high-volume PED were lower acuity (Emergency Severity Index level 4 or 5), with approximately 55,000 annual visits and an admission rate of 8.4%.^17^ This compares to a national PED median number of annual visits of approximately 25,000, with 46.4% being lower acuity, and an average admission rate of 15 %.^20^ Our PED had overall higher volumes, a higher percentage of lower acuity visits, and lower admission rates compared to the other PEDs. The high proportion of lower acuity patients seen in our ED is due to poor access to pediatric urgent care in the area and limited pediatrician office hours. Therefore, by decreasing the burden of these lower acuity visits on wait times in the ED, there is an impact on overall LOS and potentially on the quality of care provided to all our patients, especially those of higher acuity. We hypothesize that rapid assessment zone implementation in similar settings (high volume, high proportion of low-acuity patients) would prove to be the most effective, as is demonstrated with our PED.

We did not look at the impact of the COVID-19 pandemic on our ED throughput. In general, there were fewer pediatric ED visits nationally during the pandemic; LOS was prolonged compared to pre-pandemic times.^26^–^31^ This corresponded with a greater proportion of high-acuity visits, as well as increased sanitation measures, staffing shortages, requirements for use of personal protective equipment, and other factors that increased the time needed to deliver safe and effective care. Given the generally low acuity of COVID-19 symptoms in pediatric populations, many COVID-19–related visits were likely allocated to a rapid assessment zone. Therefore, further research is needed to assess the effect of the pandemic on LOS and post-pandemic recovery in PEDs with and without a rapid assessment zone. Additional research may be done to assess the impact of improved ED throughput on rates of nosocomial transmission.

## LIMITATIONS

One limitation of this study is the inability to control for residual confounding factors that may have influenced ED throughput. Factors that may have changed over the course of the study period, which were not explicitly tracked, include staffing volume, charting procedures, or general ED operations. Moreover, increases in patient volume and changes in the type of acuity over this six-year period may have also influenced results. Other limitations of note include being a single-site study, and we did not collect data on multiple other outcomes assessed in the literature for ED throughput, such as rates of leaving without being seen or leaving before treatment completion. Further studies may choose to include these additional parameters to encompass a broader scope of ED throughput analysis.

## CONCLUSION

In this retrospective operational study, implementing a rapid assessment zone in an urban pediatric emergency department significantly decreased overall length of stay. This change differed by visit acuity, with those with the lowest levels of acuity benefiting the most with a more sustained and larger decrease in LOS over time. In emergency departments with high volumes of low-acuity visits, we believe that implementation of a rapid assessment zone model is potentially useful in improving ED throughput. However, further research is needed to assess the effect of the COVID-19 pandemic on LOS and post-pandemic recovery in our pediatric. ED.

## Figures and Tables

**Figure 1:**
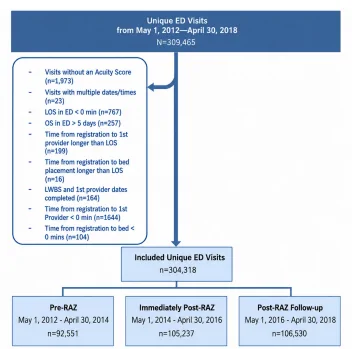
Inclusion and exclusion study criteria for ED visits in a retrospective cohort study of rapid assessment zone implementation in a pediatric emergency department. *ED*, emergency department; *LOS*, length of stay; *LWBS*, left without being seen; *RAZ*, rapid assessment zone.

**Figure 2:**
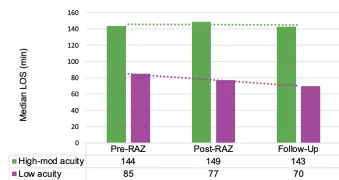
Length of stay by acuity level over study periods in a retrospective cohort study of rapid assessment zone implementation in a pediatric emergency department. *ED*, emergency department; *LOS*, length of stay; *min*, minute; *mod*, moderate; *RAZ*, rapid assessment zone.

**Figure 3:**
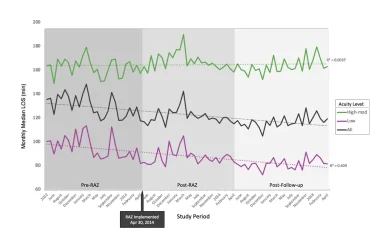
Length of emergency department stays by month and acuity during pre-rapid assessment zone, post-rapid assessment zone, and post-follow-up rapid assessment zone study periods in a retrospective cohort study of rapid assessment zone implementation in a pediatric emergency department. *LOS*, length of stay; *min*, minute; *RAZ*, rapid assessment zone.

**Table 1:** Rapid assessment zone inclusion and exclusion criteria for triage disposition.

Inclusion criteria	Exclusion criteria
Abdominal pain Cellulitis Chest pain (non-cardiac patient) Congestion Constipation Cough CPS/CSA Croup without stridor Dental complaints Diarrhea (non-bloody) Difficulty breathing Dysuria Ear pain Extremity pain without deformity (swelling OK) Eye drainage Fever Foreign body (ingested or nose/ear) Headache Head injury Lacerations (simple, must visualize) Nosebleeds Rashes (not petechial) Simple febrile seizure (< 1 in 24 hours, < 20 minutes duration) Sore throat STI check Syncope (no cardiac history) Testicular pain Vomiting	< 60 days old with fever/low temperature Asthma score > 5 Amputations Bilious emesis C-collar and backboard Ingestion Limping with fever Medically complex patients Nail bed injuries Obvious incision and drainage needed Open fractures Petechial rash Pre-arrival from specialty clinics Pregnant patients Retractions or respiratory distress Sedation Swallowed magnet or battery

*CPS*, child protective services; *CSA*, child sexual assault; *RAZ*, rapid assessment zone; *STI*, sexually transmitted infections.

**Table 2:** Changes in throughput-related outcomes length of stay, registration to bed, and registration to first clinician contact) over study periods during pre-rapid assessment. zone, post-rapid assessment zone, and post-follow-up study periods in a pediatric emergency department.

Outcome	Study Period			*P* value

	Pre-RAZ n=92,551	Post-RAZ n=105,237	Post-follow-up n=106,530	
LOS (minutes)	110 [72, 165]	101 [64, 156]	94 [59, 148]	< .001
Registration to bed (minutes)	5 [3, 10]	6 [3, 16]	5 [3, 11]	< .001
Registration to first clinician contact (minutes)	15 [8, 30]	15 [8, 31]	13 [7, 27]	< .001

Data are reported as median [25^th^ percentile, 75^th^ percentile]. *P* values are based on Kruskal-Wallis comparison of medians and Wilcoxon rank-aum test with Bonferroni correction for pairwise comparisons.

*LOS*, length of stay; *RAZ*, rapid assessment zone.
